# I–V Characteristics and Electrical Reliability of Metal–Si_x_N_y_–Metal Capacitors as a Function of Nitrogen Bonding Composition

**DOI:** 10.3390/mi16060615

**Published:** 2025-05-24

**Authors:** Tae-Min Choi, Eun-Su Jung, Jin-Uk Yoo, Hwa-Rim Lee, Songhun Yoon, Sung-Gyu Pyo

**Affiliations:** School of Integrative Engineering, Chung-Ang University, 84, Heukseok-ro, Dongjak-gu, Seoul 06974, Republic of Korea; c79411@gmail.com (T.-M.C.); eunsuj@cau.ac.kr (E.-S.J.); wlsdnr5771@naver.com (J.-U.Y.); ghkfla0725@naver.com (H.-R.L.); yoonshun@cau.ac.kr (S.Y.)

**Keywords:** MIM, capacitors, metal–insulator–metal, electrical performance, Si_x_N_y_, leakage current density

## Abstract

In this study, we analyzed the electrical characteristics of metal–insulator–metal (MIM) capacitors fabricated with reference to insulator (Si_x_N_y_) thickness and deposition condition. Si_x_N_y_ thicknesses of 650 Å, 500 Å, and 400 Å were used with four different conditions designated as MIM (N content 1.49), NEWMIM (N content 28.1), DAMANIT (N content 1.43), and NIT (N content 0.30), deposited by controlling gas flow and RF power as a function of N content. Capacitor characteristics were evaluated mainly in terms of the relationship between leakage current and breakdown voltage (BV). Current–voltage (I–V) characterizations revealed that a higher N–H/Si–H ratio effectively suppressed trap-assisted leakage conduction and enhanced dielectric robustness under high-field stress. Among the tested conditions, the NEWMIM process demonstrated the most favorable electrical performance with highest N contents. The MIM and NEWMIM conditions proved most effective among the evaluated processes, achieving sufficient BV values (>20 V) for reliable MIM capacitor operation and proposing a process optimization framework for integrating medium-density Si_x_N_y_–based MIM capacitors (2 fF/µm^2^) with sufficiently high BV values in the future.

## 1. Introduction

Metal–insulator–metal (MIM) capacitors have garnered significant interest in radio frequency (RF) and analog mixed-signal integrated circuit (IC) applications owing to their low electrode resistance, low parasitic capacitance, high charge mobility, and excellent energy storage capabilities [1,2,3,4,5,6,7]. They have been widely utilized as compact energy storage elements in various configurations, including analog ICs, RF circuits, high-power microprocessor units (MPUs), and DRAM applications [8,9,10,11,12]. As RF devices operate at increasingly higher frequencies, achieving a high capacitance per unit area becomes critical for minimizing chip area and enhancing circuit performance [13,14,15].

To meet these demands, careful optimization of design parameters including electrode materials, insulator selection, dielectric thickness, and deposition methods is essential [16,17]. Among these factors, the dielectric material plays a pivotal role, directly impacting capacitance density (CD), leakage current density (LCD), and device reliability [18,19,20,21]. In particular, precise evaluation of current–voltage (I–V) characteristics is indispensable for understanding intrinsic dielectric behavior and ensuring long-term reliability under practical bias conditions. The capacitance density is theoretically determined by the dielectric constant k and insulator thickness d as expressed by Equation (1) [1,22]:

(1) $$C=\frac{k\varepsilon_{0}A}{d}\to \frac{C}{A}=\frac{k\varepsilon_{0}}{d}$$

where C is the capacitance (F); ε_0_ is the permittivity of vacuum (8.854 × 10^−12^/m); and A is the electrode area. Although decreasing the insulator thickness can effectively increase CD, previous studies have reported that the relative permittivity (k) tends to decrease as thickness is reduced, especially in high-k materials [16,23,24,25]. Therefore, achieving high capacitance density while maintaining dielectric strength at a reduced thickness remains a significant challenge.

The use of high-k materials can partially mitigate this issue [24,26,27]; however, their implementation demands significant investment in fabrication facilities. In contrast, optimizing the deposition conditions of medium-k materials such as Si_x_N_y_ (k ≈ 7) provides a cost-effective and CMOS–compatible strategy to enhance capacitance without extensive process changes [28,29].

In our previous study, we focused on evaluating the capacitance–voltage (C–V) characteristics of Si_x_N_y_–based MIM capacitors, particularly examining the impact of silane surface treatment and dielectric thickness variation on capacitance uniformity and voltage/temperature coefficient behaviors [30]. While that work provided insights into dielectric uniformity and capacitance stability, systematic evaluation of leakage current behavior and dielectric breakdown mechanisms as a function of deposition conditions remained unexplored.

Building upon our previous findings, the present study extends the investigation to the I–V characteristics of Si_x_N_y_ films, with a particular focus on optimizing leakage current suppression and breakdown voltage enhancement through deposition condition control.

We aim to establish a process optimization framework for medium-k Si_x_N_y_–based MIM capacitors by systematically analyzing the relationship between deposition conditions and electrical reliability. We fabricated MIM capacitors with varying Si_x_N_y_ thicknesses and deposition conditions. Detailed I–V measurements were conducted to establish correlations between deposition parameters, N content (N–H/Si–H), and leakage current behavior, providing a feasible approach for developing high-reliability, medium-k MIM capacitors suitable for next-generation RF and analog IC applications.

## 2. Materials and Methods

**Figure 1:**
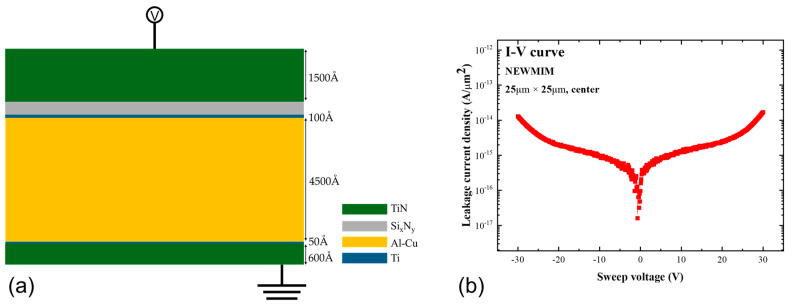
(**a**) Schematic of fabricated MIM structure and (**b**) variation in leakage current density with sweep voltage for NEWMIM condition capacitor.

The bottom electrode of the MIM capacitor was prepared using Ti (100 Å)/Al–Cu (4500 Å)/Ti (50 Å)/TiN (600 Å) wiring, and the top electrode was prepared using TiN (1500 Å). The insulator was Si_x_N_y_. The capacitor fabrication process was as follows: bottom electrode deposition → bottom electrode scrub → insulator deposition → top metal deposition → MIM PH → MIM → TOP METAL etching → ((CH_3_)_4_NOH:H_2_O, Sigma Aldrich, Incheon, Republic of Korea) cleaning 1 → MIM asher → ((CH_3_)_4_NOH:H_2_O) cleaning 2 → insulator etching → ACT 935 (wet PR strip solution including amine, Merck, Seoul, Republic of Korea) → UVAS. MIM ET was performed using the endpoint detection method. The fabricated MIM capacitor structure is shown in Figure 1a. The Si_x_N_y_ insulator was deposited using plasma-enhanced chemical vapor deposition (PECVD), with each condition achieved by precisely adjusting the gas flow ratios (SiH_4_, NH_3_, and N_2_) and RF power. To investigate the effect of nitrogen incorporation on electrical performance, we designed four deposition conditions (MIM, NEWMIM, DAMANIT, and NIT) based on the measured N contents, which were expressed as the N–H/Si–H bonding ratio. The N–H/Si–H ratios for each condition were 1.49 for MIM, 28.1 for NEWMIM, 1.43 for DAMANIT, and 0.30 for NIT. These values were quantitatively determined using X-ray Photoelectron Spectroscopy (XPS, Thermo Fisher, Waltham, MA, USA) during the process setup stage to evaluate the relative bonding composition in the Si_x_N_y_ films. Three thicknesses (650 Å, 500 Å, and 400 Å) were applied to evaluate thickness-dependent behavior under each deposition condition.

The resulting film properties, including intrinsic stress and deposition uniformity, are summarized in Table 1(a), while the specific process parameters are presented in Table 1(b). To confirm process reliability, Within Wafer and Wafer-to-Wafer uniformity were verified to be within 3%, ensuring reproducibility across all samples.

The current–voltage (I–V) characteristics of the fabricated capacitors were measured using an HP4156 semiconductor parameter analyzer (Agilent, Santa Clara, CA, USA) in manual mode, with consideration given to the instrument’s resolution. The measurement conditions are summarized in Table 2.

## 3. Results and Discussion

The electrical characteristics of the MIM capacitors were determined based on I–V measurements. A total of 12 conditions, involving four Si_x_N_y_ deposition conditions and three insulator thicknesses, were employed as shown in Table 3. The capacitor size was fixed to 25 × 25 µm^2^ to ensure consistency and eliminate the influence of areal scaling on leakage current levels, and the measurements were recorded at the top, center, and bottom of the wafer under each condition. Figure 1b shows the I–V curve of the MIM capacitor with the 650 Å insulator prepared using the NEWMIM condition. The leakage behavior was symmetrical at 0 V, and this was observed for all the capacitors, regardless of the Si_x_N_y_ thickness or condition. Table 3 summarizes the LCD values for each Si_x_N_y_ thickness and condition. The voltage was measured to be 3.7 V with a 10% margin, targeting the typical operating voltage of 3.3 V for MIM capacitors.

When fabricated using the MIM, NEWMIM, and DAMANIT conditions, the capacitors exhibited LCD values of less than 1 fA/µm^2^ (@3.7 V) for all insulator thicknesses. For the NIT condition, the capacitor with the 650 Å insulator thickness achieved an LCD of less than 1 fA/µm^2^ (@3.7 V), while those with the 500 Å and 400 Å insulator thicknesses exhibited LCD values greater than 1 fA/µm^2^ (@3.7 V).

**Figure 2:**
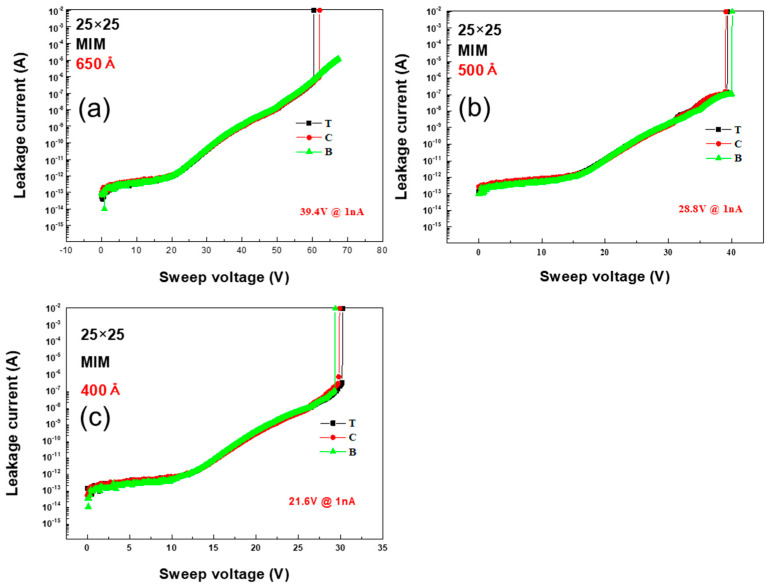
Variation in leakage current with sweep voltage for MIM condition capacitors with Si_x_N_y_ layer thicknesses of (**a**) 650 Å, (**b**) 500 Å, and (**c**) 400 Å; the corresponding BV values are 39.4 V, 28.8 V, and 21.6 V, respectively.

To characterize the BV, voltage sweeps up to 70 V were conducted for each Si_x_N_y_ thickness and condition, and the BV was the voltage at which the leakage current reached 1 nA. Figure 2 shows the BV measurement results at the top, center, and bottom of the wafer for the capacitor fabricated using the MIM condition. Figure 2a–c present the results for the wafer thicknesses of 650 Å, 500 Å, and 400 Å, respectively; the BV values were 39.4 V, 28.8 V, and 21.6 V for each thickness with the center as the reference. Figure 2 shows that for every thickness, the leakage current behavior was the same at the top, center, and bottom of the wafer and that the BV decreased with thickness.

**Figure 3:**
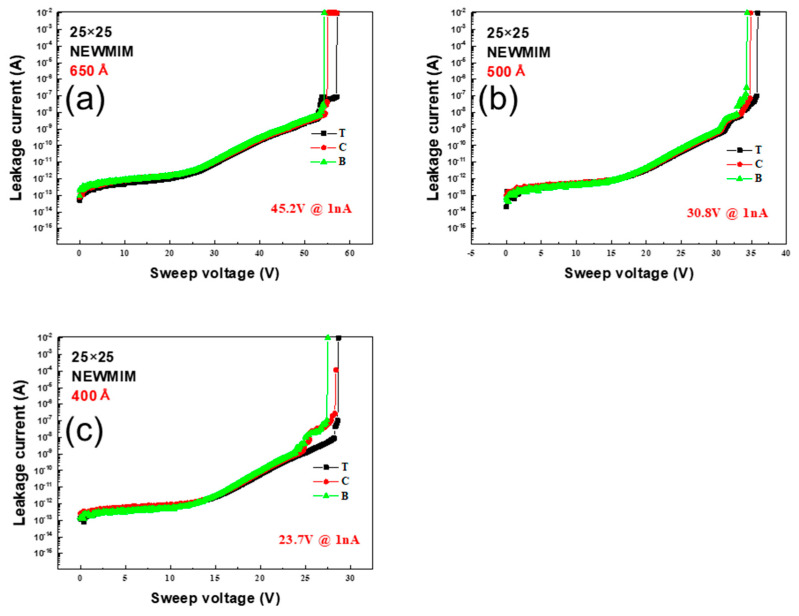
Variation in leakage current with sweep voltage for NEWMIM condition capacitors with Si_x_N_y_ layer thicknesses of (**a**) 650 Å, (**b**) 500 Å, and (**c**) 400 Å; the corresponding BV values are 45.2 V, 30.8 V, and 23.7 V, respectively.

The BV measurements for the NEWMIM condition are shown in Figure 3. Specifically, Figure 3a–c present the results for the wafer thicknesses of 650 Å, 500 Å, and 400 Å, respectively; the BV values were 45.2 V, 30.8 V, and 23.7 V for each thickness with the center as the reference. As with the MIM condition, the leakage current behavior for every thickness was the same at the top, center, and bottom of the wafer, although the BV values were slightly higher.

**Figure 4:**
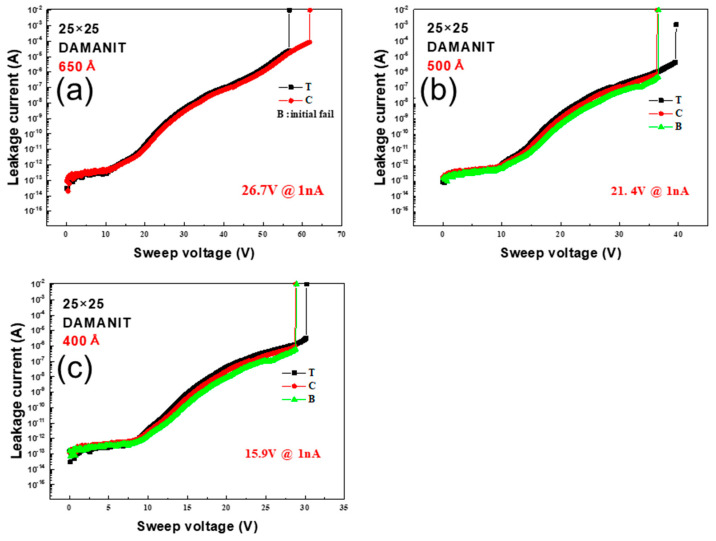
Variation in leakage current with sweep voltage for DAMANIT condition capacitors with Si_x_N_y_ layer thicknesses of (**a**) 650 Å, (**b**) 500 Å, and (**c**) 400 Å; the corresponding BV values are 26.7 V, 21.4 V, and 15.9 V, respectively.

The BV measurements for the DAMANIT condition are shown in Figure 4a–c, which present the results for the wafer thicknesses of 650 Å, 500 Å, and 400 Å, respectively; the BV values were 26.7 V, 21.4 V, and 15.9 V for each thickness with the center as the reference. As with the MIM condition, the leakage current behavior for every thickness was the same at the top, center, and bottom of the wafer; however, the BV values were slightly lower.

**Figure 5:**
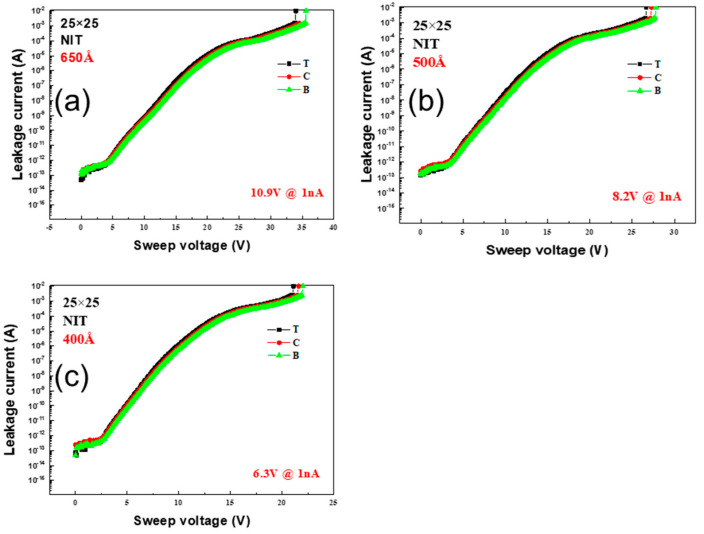
Variation in leakage current with sweep voltage for NIT condition capacitors with Si_x_N_y_ layer thicknesses of (**a**) 650 Å, (**b**) 500 Å, and (**c**) 400 Å; the corresponding BV values are 10.9 V, 8.2 V, and 6.3 V, respectively.

Figure 5 shows the BV measurements for the NIT condition. Specifically, Figure 5a–c depict the results for the wafer thicknesses of 650 Å, 500 Å, and 400 Å, respectively; the BV values were 10.9 V, 8.2 V, and 6.3 V for each thickness with the center as the reference. As with the MIM condition, the leakage current behavior for all the thicknesses was the same at the top, center, and bottom of the wafer; however, the BV values were the lowest among all the conditions.

**Figure 6:**
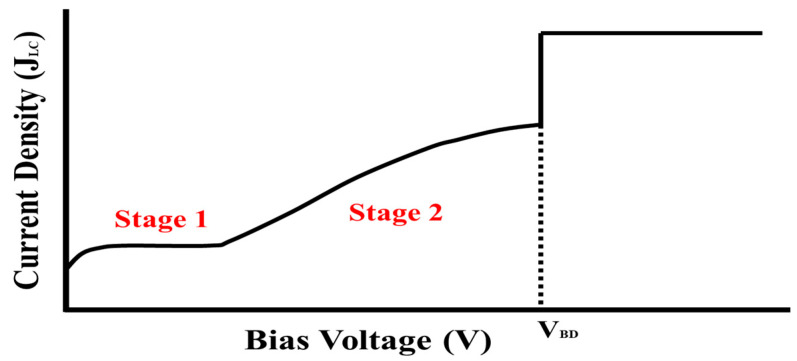
Schematic representing typical current–voltage behavior of MIM capacitors.

The general I–V characteristics of the MIM capacitor are illustrated in Figure 6. In the initial stage, electrons are injected across the energy barrier between the electrode and dielectric or are captured at trap sites under an applied electric field. Conduction behavior is governed by the Poole–Frenkel mechanism, wherein thermally activated electrons induce a linear relationship between J (Current)/E (Electric Field) and √E [31,32]. As the bias increases, the available trap sites are progressively saturated, leading to a transition into Stage 2. In this regime, enhanced field emission and detrapping phenomena become dominant [33], resulting in a rapid escalation of current that signifies the onset of dielectric degradation, and when a critical bias is applied, the band bending is amplified, and the tunneling effect, which is minimal in Stages 1 and 2, is maximized, resulting in physical and electrical breakdown [5]. In this study, the breakdown voltage (BV) of the MIM capacitors was operationally defined as the voltage at which the leakage current exceeded 1 nA. Accordingly, the BV corresponds to the bias voltage at which the transition to Stage 2 commences in Figure 6.

The BV values of the MIM capacitors for every Si_x_N_y_ layer thickness and deposition condition are summarized in Table 4. For all conditions, the BV decreased with thickness. Among the four conditions, the NEWMIM condition provided the best BV value for a given thickness; for the remaining conditions, the BV values decreased in the order of MIM, DAMANIT, and NIT. In MIM capacitors, the leakage current exhibits a strong inverse dependence on dielectric thickness, primarily due to enhancement of the internal electric field as the thickness decreases. A thinner dielectric layer leads to higher field-assisted emission processes such as Poole–Frenkel conduction, thereby resulting in an exponential increase in leakage current density [31,34].

**Figure 7:**
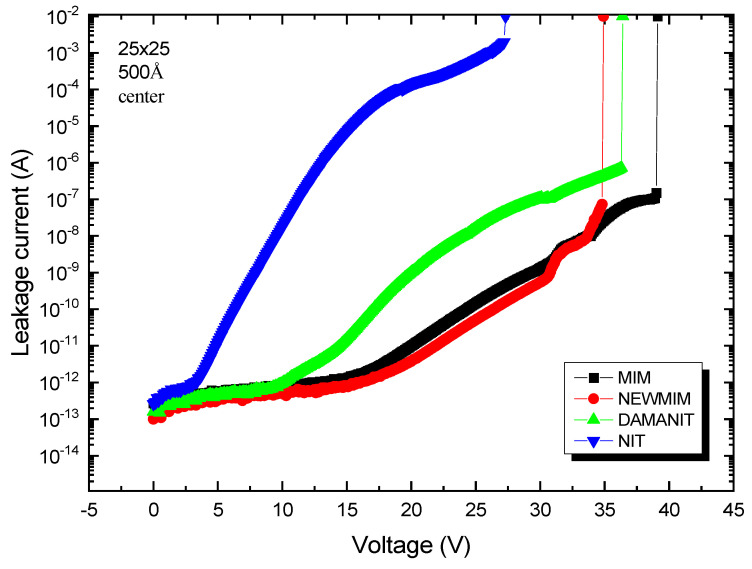
Variation in leakage current with sweep voltage for MIM capacitors fabricated using different conditions with a Si_x_N_y_ layer thickness of 500 Å.

To interpret the BV values based on the condition, the N–H/Si–H ratio for each condition is shown in Table 1. Further, Figure 7 shows the variation in leakage current with the bias for each condition at the same thickness (500 Å). To interpret the BV values based on material conditions, the N–H/Si–H ratio for each condition is summarized in Table 1. Figure 6 presents the variation in leakage current with applied bias for each condition at a fixed thickness of 500 Å. A higher N–H/Si–H ratio correlates with a lower leakage current density (LCD) and higher breakdown voltage (BV). Among the samples, the NEWMIM condition exhibited the highest N–H/Si–H ratio at 28.1, whereas the NIT condition showed the lowest value of 0.30. Correspondingly, as shown in Figure 7, the leakage current in Stage 2 was lowest for NEWMIM and highest for NIT. This trend is attributed to the difference in trap density within the dielectric.

Several studies have reported that for MIM capacitors employing amorphous Si_x_N_y_ films prepared by plasma-enhanced chemical vapor deposition, an increased N–H/Si–H bonding ratio (i.e., higher N content) leads to a reduction in leakage current [13,35,36,37,38,39]. In Si_x_N_y_ films, a higher N–H bond concentration effectively reduces the trap density within the dielectric. As a result, trap-assisted tunneling and field-enhanced detrapping are suppressed, leading to a slower increase in leakage current under high electric field stress. Conversely, films with higher trap densities exhibit enhanced trap filling and detrapping, facilitating additional leakage pathways and causing a steeper escalation in leakage current. The replacement of weaker Si–H bonds with stronger Si–N bonds suppresses the formation of dangling bonds, thus mitigating trap-assisted conduction mechanisms. This is consistent with the Poole–Frenkel framework, where lower trap densities diminish field-enhanced carrier emission, contributing to higher breakdown voltages. Thus, increasing the N–H bonding ratio is an effective strategy to improve the high-field leakage characteristics of Si_x_N_y_–based MIM structures. Under this assumption, our experiments showed that Si_x_N_y_ thin films fabricated under NEWMIN and MIM conditions fulfilled high BV values of more than 20 V even at a thickness of 400 Å. Furthermore, a comparison across different film thicknesses highlights that under high nitrogen incorporation (as in the NEWMIM and MIM conditions), the BV values at 400 Å exceeded those of 500 Å films fabricated under lower nitrogen conditions (DAMANIT and NIT). This observation emphasizes that dielectric performance is governed more by film quality than by thickness alone.

**Figure 8:**
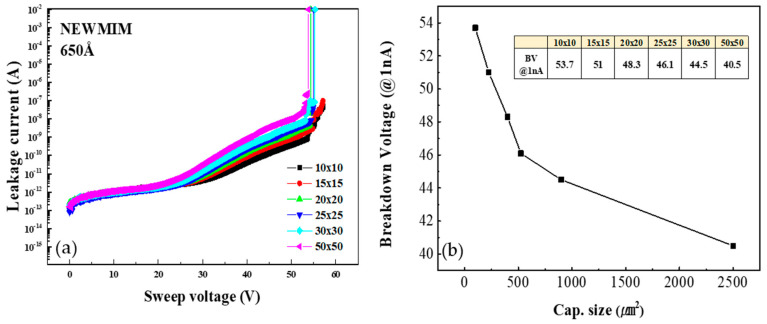
(**a**) Variation in leakage current with sweep voltage and (**b**) variation in breakdown voltage for MIM capacitors of different sizes.

**Figure 9:**
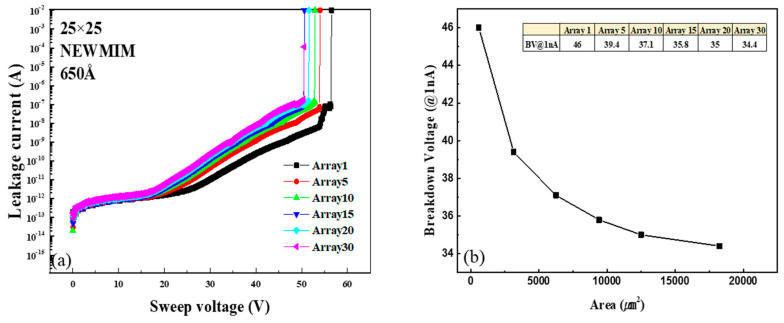
(**a**) Variation in leakage current with sweep voltage and (**b**) variation in breakdown voltage for MIM capacitors with different total capacitance areas.

To understand the relationship between the BV characteristics and capacitor size, the BV values were measured for the 10 × 10, 15 × 15, 20 × 20, 25 × 25, 30 × 30, and 50 × 50 µm^2^ capacitors fabricated using the NEWMIM condition with the 650 Å insulator thickness. Figure 8a shows the I–V curves, while Figure 8b shows the change in the BV with increasing capacitor size. The BV value is observed to decrease by up to 24.5% as the capacitor size increases. Also, to evaluate the dependence of the BV characteristics on the number of capacitor arrays, the BV values were measured for 1, 5, 10, 15, 20, and 30 arrays of the 25 × 25 µm^2^ capacitor fabricated using the NEWMIM condition with the 650 Å insulator thickness. Figure 9a shows the I–V curve, and Figure 9b shows the change in the BV value with the number of arrays. As the number of arrays (i.e., as the capacitance area) increases, the BV decreases by up to 24.7%.

The observed decrease in BV with increasing capacitor size and array number can be attributed to area-dependent reliability degradation mechanisms. As the physical area of the capacitor increases, the probability of encountering pre-existing defects within the dielectric also increases, which leads to a higher likelihood of localized dielectric breakdown. These results suggest that for large-area MIM capacitors, careful control of dielectric quality and design considerations to mitigate edge effects are essential to maintain high reliability. Nevertheless, capacitors under the NEWMIM conditions adopted in this study showed stable BV values for all areas and arrays.

## 4. Conclusions

In this study, the electrical (I–V) characteristics of MIM capacitors were evaluated with respect to Si_x_N_y_ thickness and deposition condition. To enhance the reliability of the observed electrical trends, especially the correlation between nitrogen content and breakdown behavior, I–V measurements were conducted at three distinct positions—top, center, and bottom—of the wafer for each condition and thickness. As nitrogen incorporation in films increased, breakdown voltage (BV) characteristics are enhanced. Across all insulator thicknesses, capacitors fabricated using the NEWMIM condition (N content 28.1) consistently exhibited higher BV values (@1 nA) than those fabricated under other conditions. Notably, both MIM (N content 1.49) and NEWMIM conditions achieved BV values exceeding 20 V even at a reduced thickness of 400 Å, underscoring the impact of optimized bonding environments on dielectric reliability. These results suggest that higher N contents result in improved dielectric quality through reduced trap site formation, providing a robust basis for high-field reliability in scaled MIM structures. The comprehensive I–V dataset, spanning three film thicknesses and four deposition conditions, supports a process optimization framework grounded in bonding structure control. These findings demonstrate the feasibility of developing medium-density (2 fF/μm^2^) MIM capacitors with sub-400 Å insulator layers, enabling increased capacitance density without sacrificing breakdown strength.

Beyond the immediate findings, this study suggests that lower-thickness Si_x_N_y_ layers can support high-density integration without significant loss of dielectric strength. The MIM and NEWMIM processes also appear compatible with standard CMOS BEOL thermal budgets, offering practical integration potential. Future work should explore barrier layer incorporation, stack engineering, and accelerated reliability testing to validate the long-term stability of these MIM structures.

## Figures and Tables

**Table 1:** (a) Si_x_N_y_ film properties and (b) corresponding process conditions.

**(a)**	**MIM**	**NEW MIM**	**DAMA NIT**	**NIT**
Dep. rate	~149 Å/s	~29 Å/s	~59 Å/s	88 Å/s
Within Wafer uniformity (1σ)	1.14%	1.90%	2.34%	2.77%
Wafer-to-Wafer uniformity (1σ)	1.58%	2.21%	1.05%	2.30%
Stress	−2.23 × 10^9^	−1.75 × 10^10^	−2.34 × 10^9^	
H content (N–H: Si–H)	12.7%: 8.5%	22.5%: 0.8%	10.5%: 7.3%	4.4%: 14.8%
N content (N–H/Si–H)	1.49	28.1	1.43	0.30
**(b)**	**MIM**	**NEW MIM**	**DAMA NIT**	**NIT**
Step end control	By time	By time	By time	By time
Maximum step time	4.4 s	22.8 s	11.0 s	
Endpoint selection	No endpoint	No endpoint	No endpoint	No endpoint
Pressure	Servo 4.25 Torr	Servo 4.25 Torr	Servo 4.2 Torr	Servo 4.5 Torr
RF power	690 W	690 W	420 W	425 W
Susceptor temperature	400 °C	400 °C	400 °C	400 °C
Susceptor spacing	620 mils	620 mils	550 mils	475 mils
N_2_	3800 sccm	3800 sccm	2500 sccm	4000 sccm
NH_3_	130 sccm	50 sccm	38 sccm	60 sccm
SiH_4_	260 sccm	100 sccm	110 sccm	170 sccm

**Table 2:** Measurement conditions for current–voltage characterization.

Parameter	Setting
Sweep voltage	−70 V to 70 V
Compliance	10 mA
Forcing	Top electrode
Capacitor sizes	10 × 10 µm^2^, 15 × 15 µm^2^, 20 × 20 µm^2^, 25 × 25 µm^2^, 30 × 30 µm^2^, 50 × 50 µm^2^
Measurement points	Three points (top, center, bottom)
Temperature	Room temperature

**Table 3:** Leakage current densities of MIM capacitors fabricated with different conditions and Si_x_N_y_ thicknesses.

Si_x_N_y_ Deposition Condition	Thickness (Å)	Leakage Current Density (fA/µm^2^ @ 3.7 V)
MIM	650	0.336
	500	0.72
	400	0.32
NEWMIM	650	0.656
	500	0.336
	400	0.72
DAMANIT	650	0.416
	500	0.432
	400	0.496
NIT	650	0.896
	500	1.728
	400	8.288

**Table 4:** Breakdown voltages of MIM capacitors fabricated with different Si_x_N_y_ thicknesses and conditions.

Si_x_N_y_ Deposition Condition	Thickness (Å)	Breakdown Voltages (V)
MIM	650	39.4
	500	28.8
	400	21.6
NEW MIM	650	45.2
	500	30.8
	400	23.7
DAMA NIT	650	26.7
	500	21.4
	400	15.9
NIT	650	10.9
	500	8.2
	400	6.3

## Data Availability

The original contributions presented in the study are included in the article; further inquiries can be directed to the corresponding author.
